# 528. Hospital Course of Patients Receiving Bamalanivimab: A Real World Analysis

**DOI:** 10.1093/ofid/ofab466.727

**Published:** 2021-12-04

**Authors:** Madeline Belk, Jonathan Edwards, Ali Hassoun

**Affiliations:** Huntsville Hospital, Huntsville, Alabama

## Abstract

**Background:**

Monoclonal antibodies for the outpatient treatment of the novel Coronavirus Disease 2019 (COVID-19) first received emergency use authorization from the Food and Drug Administration in November 2020. These antibodies have been associated with a reduction in emergency department visits and hospitalization through randomized controlled trials. However, modest data is available to describe the outcomes of patients who were hospitalized despite treatment. This study describes real-world outcomes concerning the treatment of COVID-19 with the first approved monoclonal antibody for COVID-19, bamlanivimab, as well as hospital courses associated with patients admitting after receiving the therapy.

**Methods:**

This single-center, retrospective study evaluated real-world data of patients treated with bamlanivimab. The primary endpoint was a composite of emergency department (ED) visits or hospitalization due to worsening COVID-19. Data was analyzed from November 23, 2020 to March 5, 2021. Descriptive statistics were used to analyze the primary endpoint. Secondary endpoints include reported symptoms 24-hours post-infusion and time to symptom resolution in days. Additionally, clinical course of patients hospitalized were analyzed and include average oxygen requirements, median length of stay, and mortality. A subgroup analysis was conducted between patients less than sixty-five years of age and those sixty-five and older.

**Results:**

619 patients received bamlanivimab during the specified timeframe. The primary endpoint occurred in 34 patients; 11 ED visits and 23 hospitalizations. Baseline characteristics of the patients hospitalized include median age 69 years (IQR 55, 74), 56.5% male, and 82.6% Caucasian. The most common risk factors for severe disease among those hospitalized were age ≥ 65 years and history of diabetes. The clinical course of hospitalized patients varied but 52.9% required nasal cannula for respiratory support and the average length of stay was 4.5 + 4.5 days. Other COVID-19 therapies included dexamethasone in 76.5% of patients and remdesivir in 47.1% of patients. There were no major differences in the subgroup analysis.

**Conclusion:**

Bamlanivimab appears to attenuate the clinical course of COVID-19 in patients who are hospitalized despite treatment.

**Disclosures:**

**All Authors**: No reported disclosures

